# Managing the Continuum of Myeloid Malignancies

**Published:** 2018-04-01

**Authors:** Andrew Artz, Jean A. Ridgeway

**Affiliations:** University of Chicago Medicine, Chicago, Illinois

## Abstract

Advanced practitioners can play an important role in assessing patients with myeloid malignancies for fitness and addressing vulnerabilities so that patients can benefit from allogeneic stem cell transplant, which remains the best option for many patients.

Myeloid malignancies remain a challenge for advanced practitioners. Their genetic profiles have become increasingly critical to diagnosis and prognosis, and patient management is changing as new therapeutics come on board. Stem cell transplant, however, remains the most effective option for many patients, although not all patients are candidates. Advanced practitioners can assess patients for fitness and address their vulnerabilities so that more patients can benefit from transplant, as discussed at JADPRO Live 2017 by Andrew Artz, MS, MD, and Jean A. Ridgeway, DNP, APN, NP-C, AOCN®, both of the University of Chicago Medicine.

## STEM CELL TRANSPLANT

"With many of the myeloid malignancies, we are still unlikely to achieve long-term control [with current medications], and allogeneic transplant remains the best option for many patients," Dr. Artz said. Transplant is recommended after achievement of remission in the following patient groups:

- Age < 60 years with intermediate-risk cytogenetics and/or molecular abnormalities
- Presence of treatment-related disease or poor-risk cytogenetics and/or molecular abnormalities
- Age ≥ 60 years after failure of induction therapy
- Presence of relapse

A growing trend is to offer transplant to older patients (≥ 60 years), assuming they are fit; in fact, the fastest growing transplant demographic is patients who are 70 or older. "Insurance coverage has been a major limitation to transplant in older patients, but that barrier is increasingly being removed. That’s where the future is heading," he predicted.

While survival at 1 and 3 years post-transplant is lower in older patients than in younger ones, the same can be said for induction and consolidation. "The bottom line is that our standard approaches are not achieving many long-term successes in older adults," he said, "so while older adults might do worse with transplant, it doesn’t mean that transplant should not be offered. It may still be of benefit, compared to standard chemotherapy."

"But there is considerable toxicity, and for the older patients, transplant is really a struggle," Dr. Artz acknowledged. "Many of you have faced this in trying to help patients navigate the illness. The patient wants to do everything to get better, but doesn’t want to do everything to get sick from it."

Taking the "gentler road" is the alternative option, and the choice between these approaches—increasingly the case as more effective therapies come on board—is hard.

## TRANSPLANT OPTIMIZATION PROGRAM

Although certain conventional exclusions for transplant still apply, it is helpful to think in terms of "chronological age vs. resiliency" and to use this information to inform candidacy for transplant and to optimize outcomes, he said. Up to half of all older transplant patients have some vulnerability that can negatively impact outcomes, and patients’ overall fitness has been consistently related to survival post-transplant.

The good news is that, when vulnerabilities are identified, fitness can often be enhanced ahead of transplant, the speakers said. They are doing this at the University of Chicago through their interdisciplinary transplant optimization program (TOP), a structured way to assess for resilience and vulnerability and to address and remediate problem areas (Table 1).

**Table 1:**
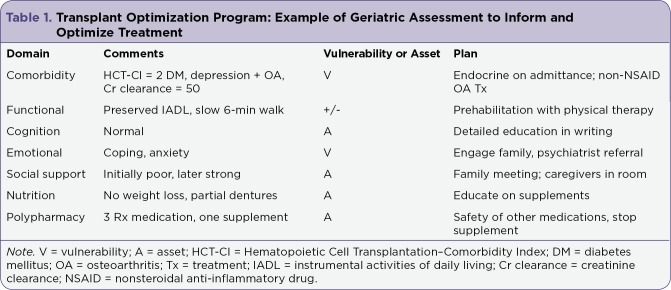
Transplant Optimization Program: Example of Geriatric Assessment to Inform and Optimize Treatment

Their center’s TOP is instituted for patients ages 50 or higher considering allogeneic or autologous transplant. The TOP team consists of the transplant physician, advanced practice nurse, geriatric oncologist, social worker, infectious disease specialist, physical therapist, dietician, and research coordinator.

"The core of TOP is nontraditional assessment to get a better understanding of resiliency vs. frailty, using a tool that’s well known in the geriatric literature—the geriatric assessment," Dr. Ridgeway said. Over the course of a half-day, the team evaluates various domains—comorbidity, function, cognition, emotional and social support, nutrition and polypharmacy—then meets to discuss their findings and to devise a maximal support plan as needed (Table 2).

**Table 2:**
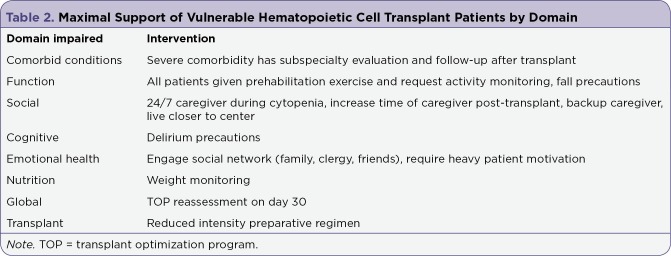
Maximal Support of Vulnerable Hematopoietic Cell Transplant Patients by Domain

"We try to tease out where the patient really is, and based on this the patient may or may not go forward to stem cell transplant. Sometimes we recommend delaying transplant, although this becomes a tenuous decision. When there is time for this, we make recommendations tailored to the patient’s needs. Sometimes we do say ’no’ to patients whose comorbidity score and current health status definitely preclude them from going forward with a transplant."

## MEASUREMENT IN THE TOP PROGRAM

For identifying comorbidities, the University of Chicago team uses the Hematopoietic Cell Transplantation–Comorbidity Index (HCT-CI), an instrument that comprises 17 different categories of organ dysfunction and that generates a score (Fred Hutchinson Cancer Research Center, 2018). The HCT-CI might, for example, identify diabetes, depression, osteoarthritis and low creatinine clearance, in which case the recommendation might be an endocrinology consult and discontinuation of nonsteroid anti-inflammatory drugs, Dr. Ridgeway said.

Physical function assessment includes a 6-minute walk test, grip strength, timed up and go measurement, and other items, with results compared to age-matched controls. The ability to perform independent activities of daily living is documented. These tests often reveal the need for prehabilitation either in or outside the home.

The patient undergoes a screening test of cognition and memory, which sometimes reveals the need for a full panel of cognitive testing. Patients are also assessed for emotional and social support (with psychiatric referral as needed), nutrition (for unintentional weight loss, a poor prognosis factor), and polypharmacy (including supplement use). To foster social support, family meetings are held and the caregivers’ full engagement is encouraged.

The transplant team uses the information gleaned from TOP to ascertain the patient’s strengths and limitations and to determine what kind of intervention will promote fitness for transplant, acknowledging that the issues can be multifactorial. "You can’t look at a single domain—maybe they aren’t walking very fast—and think that exercise or nutrition will fix everything," Dr. Artz said. "Our philosophy is that if we are going to treat you intensively, and you have some vulnerabilities, we’d best create an ecosystem that gives you some reserve. That way, if we can’t get your function where we want it, but we improve your nutrition, social support, infection, and other issues, maybe you will still be able to get through transplant because we’ve helped you create some resilience."

## WHO DISEASE CLASSIFICATIONS FOR MYELOID MALIGNANCIES IN 2017

Dr. Artz also discussed changes to the World Health Organization (WHO) disease classifications for myeloid malignancies, the key revision being a focus on genetics. "We’ve entered the genetic era in how we diagnose and classify these diseases," he said. An understanding of the patient’s tumor genetics is important for refining diagnosis, for determining prognosis, and for enriching patient selection for targeted agents. For example, the presence of a *TP53* mutation suggests a neoplasm that is possibly hereditary, could be resistant to cytotoxic chemotherapy, and might respond to a novel agent such as ibrutinib (Imbruvica).

Another change is the provisional classification of familial, or hereditary, myeloid malignancy syndromes. These fall into three categories: hematologic malignancies only (such as familial acute myeloid leukemia [AML] with mutated *CEBPA*), platelet dysfunction (such as thrombocytopenia with mutated *ETV6* or *ANKRD26*), and syndromes with additional organ systems affected (such as *GATA2* deficiency syndromes).

"There are families with multiple family members who have blood cancers and myeloid malignancies, and it’s not necessarily a coincidence. You just need to know that some hematologic malignancies are inherited and to think to test for these mutations," he told attendees.

## WHO CLASSIFICATION FOR MYELOFIBROSIS AND MYELODYSPLASTIC SYNDROMES

The WHO diagnostic criteria for overt primary myelofibrosis now requires the presence of megakaryocyte proliferation and atypia, failure to meet WHO criteria for other myeloid neoplasms, and the presence of certain clonal markers (such as JAK2). In the absence of these factors, there must be no evidence that marrow fibrosis is reactive. One other minor criterion must also be met.

"Genetic data are important in completing the classification," Dr. Artz said. "It’s not just about what it looks like under the microscope anymore."

For myelodysplastic syndromes (MDS), five essential domains are used in classification: dysplastic lineages, cytopenias, ring sideroblasts, blasts, and karyotype. Genetic panels help refine the diagnosis, and bone marrow biopsy (which remains essential) identifies dysplasia (which must be present).

## ACUTE MYELOID LEUKEMIA AND RELATED NEOPLASMS IN 2017

"AML has gotten much more complicated," Dr. Artz continued. This category now includes AML with recurrent genetic abnormalities ("provisional"), AML with myelodysplasia-related changes, therapy-related myeloid neoplasms, AML not otherwise specified, myeloid sarcoma, and myeloid proliferations related to Down syndrome.

"We find a tremendous spectrum of typical chromosome derangements and specific mutations in these patients," he said. By far the most common are *FLT3*, *NPM1*, *DNMT3A*, and *NRAS*, but more than 50 driver mutations can occur in AML (Papaemmanuil et al., 2016), and certain ones are strongly prognostic, including *FLT3-ITD*, *NPM1*, and *CEBPA*. In a study of 410 patients, those with *NPM1* or *CEBPA* mutations had significantly better relapse-free and overall survival than patients with *FLT3-ITD* mutations or patients lacking any of these three mutations (triple wild type; Schlenk et al., 2008). The concurrence of two different mutations can also alter outcomes.

"To make matters even more complicated, it’s not just about the mutation; it’s sometimes the burden of the mutation, what we call the allelic ratio or mutant fraction," he said. "So when you do some of these assays, some will just say ’mutated,’ but more and more frequently you are going to get a number that is going to say 20% or 30% or 50%, or 0.5 or 0.6. These numbers are prognostically relevant" (Gale et al., 2008).

## ’THE’ YEAR FOR AML THERAPY

Dr. Ridgeway described newly approved agents for AML, including midostaurin (Rydapt), daunorubicin/liposomal cytarabine (Vyxeos; CPX-351), gemtuzumab ozogamicin (Mylotarg), and enasidenib (Idhifa), and the investigational drugs vosaroxin, vadastuximab, venetoclax (Venclexta), selinexor, and hypomethylators.

"The year 2017 has been ’the’ year for AML drug development," Dr. Ridgeway said. In April 2017, the FLT3 inhibitor midostaurin became the first drug to be approved for AML in the US since the year 2000. In the RATIFY trial of 764 AML patients with *FLT3-ITD* or *TKD* mutations, 5-year overall survival was 51% for patients receiving 7 + 3 plus midostaurin vs. 43% with 7 + 3 alone (Stone et al., 2017). Midostaurin is approved for adults with newly diagnosed *FLT3*-mutated AML.

Intravenous liposomal cytarabine and daunorubicin is a very effective drug that selectively hones in on malignant cells in the bone marrow. CPX-351 is approved for adults with newly diagnosed therapy-related AML or AML with changes indicative of MDS. In the pivotal trial, median overall survival was 9.6 months with the new compound vs. 5.9 months with conventional 7 + 3 (Lancet et al., 2016). CPX-351 is now the new standard for older high-risk AML patients who are candidates for intensive therapy, she said.

Gemtuzumab ozogamicin was approved in September 2017 for adults and children with newly diagnosed or relapsed AML. Myeloid suppression and hepatic toxicities are side effects to watch for with this agent.

For the 10% or so of patients with *IDH2*-mutated relapsed/refractory AML, the new oral targeted agent is enasidenib. In the pivotal trial, the overall response rate was 40.3%, complete remissions were achieved by 19.3%, and complete responders had a median overall survival of 19.7 months (Stein et al., 2017). Clinicians should be alert to the potential for differentiation syndrome and hepatic toxicity with enasidenib.

"There are a lot of other drugs being studied in AML, many targeting unique markers, both as single agents and in combinations," Dr. Ridgeway continued. "We are beginning to shift our goals of therapy from simply ’treatment’ to ’cure.’ "

## ’CLASSIC’ MYELOPROLIFERATIVE NEOPLASMS

Dr. Ridgeway described the classic myeloproliferative neoplasms (MPNs) as comprising two main groups: *BCR-ABL1*–positive and *BCR-ABL1*–negative. Under the former fall polycythemia vera (PV), essential thrombocythemia (ET), and primary myelofibrosis and post-PV/ET myelofibrosis. The *BCR-ABL1*–positive group includes patients with chronic myeloid leukemia (CML).

The JAK-STAT pathway is necessary for growth and differentiation and is involved in inflammatory cytokine signaling and immune regulation. In 2011, the first JAK2 inhibitor, ruxolitinib (Jakafi), was approved for intermediate- or high-risk myelofibrosis. Other JAK2 inhibitors are also in development. Ruxolitinib can effectively control the constitutional symptoms associated with myelofibrosis, she said.

Myelosuppression, cytopenias, and diarrhea are the anticipated side effects of ruxolitinib. To maximize efficacy and minimize toxicity, clinicians can individualize dosing based on platelet count. To prevent symptom flares, practitioners should taper slowly when discontinuing therapy, Dr. Ridgeway advised.

For CML, the list of tyrosine kinase inhibitors has grown to include imatinib (Gleevec), nilotinib (Tasigna), and dasatinib (Sprycel) for frontline use, and nilotinib, dasatinib, bosutinib (Bosulif), ponatinib (Iclusig), and omacetaxine (Synribo) for second- and third-line use, along with several agents in other classes. The hope is that these targeted agents may be curing some patients; studies are addressing the possibility of discontinuing treatment in subgroups of patients with stringent molecular remissions, Dr. Ridgeway indicated.
